# Natural History of Radiographic First Metatarsophalangeal Joint Osteoarthritis: A Nineteen‐Year Population‐Based Cohort Study

**DOI:** 10.1002/acr.24015

**Published:** 2020-08-31

**Authors:** Catherine Bowen, Lucy Gates, Peter McQueen, Maxine Daniels, Antonella Delmestri, Wendy Drechsler, David Stephensen, Michael Doherty, Nigel Arden

**Affiliations:** ^1^ University of Southampton Southampton UK; ^2^ Oxford Health NHS Trust Oxford UK; ^3^ University of Oxford Oxford UK; ^4^ King’s College London UK; ^5^ Canterbury Christ Church University Canterbury UK; ^6^ University of Nottingham Nottingham UK; ^7^ University of Oxford, Oxford, UK, and University of Southampton Southampton UK

## Abstract

**Objective:**

To assess the long‐term prevalence, natural history, progression, and incidence of radiographic first metatarsophalangeal (MTP) joint osteoarthritis (OA).

**Methods:**

A longitudinal cohort design was used in which radiographic OA at the first MTP joint was investigated in participants from the Chingford 1,000 Women Study at year 6 (1995) and year 23 (2013–2015). Radiographic features of osteophytes (OPs) and/or joint space narrowing (JSN) at the first MTP joint were scored according to a validated foot atlas. Natural history was determined by the change in prevalence, incidence, progression, and worsening of OA in the first MTP joint.

**Results:**

Complete case‐matched foot radiographic data were available for 193 of the women currently enrolled in the study (mean ± SD age 75.7 ± 5.2 years [range 69–90 years]). At the level of the first MTP joint, prevalence of OA at year 6 was 21.76% in the left and 24.35% in the right; at year 23, it was 23.83% in the left and 32.64% in the right. Over the 19‐year period, 13.5% of the women developed incident OA in the right first MTP joint and 8.3% in the left. Both progression and worsening of OA were more evident for OPs and in the right first MTP joints.

**Conclusion:**

In this study of the natural history of radiographic first MTP joint OA, which to our knowledge is the longest study to date, the prevalence and incidence of first MTP joint OA increased over a 19‐year period. Progression and/or worsening of OA at the first MTP joint over time appears to be driven by OP development rather than JSN, which suggests a biomechanical cause.

## INTRODUCTION

There is increasing evidence from a growing number of population cohort studies that foot osteoarthritis (OA) is common, especially in older adults with foot pain 1, 2, 3, 4. The UK population prevalence of symptomatic radiographic foot OA overall has been estimated at 17% and, for the individual joint level, at 8% for the first metatarsophalangeal (MTP) joints in adults ages >50 years 2. Foot pain has been reported to affect between 7% and 13% of adults in the US (age 30–100 years) 5, and in the UK, 10% report disabling foot pain 6. Structural foot OA has also been linked to outcomes such as foot pain, restricted activity, lower quality of life 2, 7, 8, 9, 10, and increased visits with general practitioners 11, 12.

**Significance & Innovations**

- Investigation of osteoarthritis (OA) over time presents a major evidence gap in the field of foot and ankle OA. This study is the first to investigate OA over a 19‐year period, confirming that the prevalence of radiographic OA within the first metatarsophalangeal (MTP) joint in older women increases over time.
- Although previously observed in hand OA, the discordance in right‐left findings for osteophyte development and joint space narrowing observed in the first MTP joint over time has not been described previously.
- Findings from this study implicate biomechanical factors as a cause in the development of first MTP joint OA. Further work is required to investigate potential biomechanical risk factors for the development and progression of first MTP joint osteophytes and OA.

Studies reporting on the incidence of OA of the knee and hip are becoming more evident 13, 14, 15. Slow development of radiographic knee OA, stable progression, and improvement over long periods have also been reported 16, 17. In contrast to knee and hip OA, there are very few studies on the epidemiology and management of foot and ankle OA. Evidence relating to foot OA is often reported from cross‐sectional study designs, and comparatively little is known about potential changes in foot joints over time 9, 10. A recent study investigating self‐reported foot pain over 18 months reported little symptomatic changes in 3 different foot OA phenotypes determined at baseline 18.

The lack of foot‐specific longitudinal data, notably the lack of valid measurement criteria for foot OA progression over time, has been highlighted as a limitation in the current body of knowledge on radiographic foot OA, which makes investigations evaluating clinical interventions challenging 2, 9. Thus, further longitudinal investigation of incidence, progression, and natural history of foot OA is warranted in order to target preventive therapies and reduce known modifiable risk factors for both the incidence and progression of foot OA 9, 10. One US cohort study, reporting on findings from the community‐based Clearwater Osteoarthritis Study, has presented foot OA incidence data 19. Over a period of 7 years, 364 participants (25%) and 404 participants (27%) developed structural left and right, first MTP joint OA, respectively 19. Unfortunately, a key limiting factor for the generalizability of those findings is that the sample was selected according to the presence of hindfoot valgus, and thus the data are not representative of the general population.

The Chingford 1,000 Women Study is a 25‐year longitudinal population cohort, which provides a unique opportunity to investigate foot OA over time. The aim of this study was to assess the long‐term natural history of first MTP joint OA by observing prevalence, incidence, progression, and worsening of radiographic foot OA in this well‐described, population‐based cohort of older women in the UK over a 19‐year period.

## PATIENTS AND METHODS

#### Participant selection

The Chingford 1,000 Women Study was established in 1989 to study the health of women in midlife. It involves a prospective cohort that originally comprised 1,003 women ages 45–64 years from a general practice in Chingford, London, UK. Participants have been followed annually since 1989 and are described in detail elsewhere 20, 21, 22, 23.

All participants retained within the Chingford 1,000 Women Study who had complete radiographs of the foot at year 6 (1995) and also at year 23 (2013–2015) were included in this study. Participants with foot radiographs that were damaged and/or unreadable from either year or were missing from year 6 were excluded (n = 25). All included participants provided informed written consent. Full ethics approval was granted by the Waltham Forest and Redbridge local research ethics committee (reference number: LREC R&WF 96). Approval for The Chingford 1,000 Women Year‐23 Foot Study was granted by the National Research Ethics Service Committee, South Central–Oxford A, in May 2013 (REC number: 84131).

#### Data collection

Assessments for year 23 took place during a single appointment at the Silverthorne Medical Centre, Chingford, UK. On each occasion, the same consultation rooms and facilities were utilized. Foot radiographs were obtained in 1995 at the InHealth NHS Stratford site, UK. Due to a change in contract, all follow‐up radiographs in 2013–2015 were undertaken at Holly House Private Hospital in Chigwell, UK. A standard operating procedure for foot radiographs had been drawn up a priori, and for both radiography sites, consensus meetings with the radiographers were held to ensure consistency between sites. Data collection for year 6 had been carried out by the previous study investigators at the Stratford site. All foot radiographs were performed at both time points after the demographic assessments on the same day or as close as possible.

#### Assessment of participant characteristics and radiographic foot OA

General characteristic data including age, weight, and height were recorded by an experienced registered nurse (M. Daniels) at both data collection points. All foot radiographs obtained at year 23 included a single dorsoplantar view of both feet and separate lateral views of both feet according to a standardized protocol 24, 25. Foot radiographs were taken barefoot and partially weight‐bearing and were available in electronic format. The radiographic films were reviewed by a consultant radiologist at the InHealth NHS Stratford radiology unit or at the Holly House Hospital Department of Radiology for any radiographic red flags or clinically significant abnormalities.

Radiographic features of osteophytes (OPs) and/or joint space narrowing (JSN) at each first MTP joint were scored according to the La Trobe Foot Atlas 24. The atlas uses a 4‐point scale of 0, 1, 2, and 3 to score OPs (0 = absent, 1 = small, 2 = moderate, 3 = severe) and JSN (0 = none, 1 = definite, 2 = severe, 3 = bone‐on‐bone ≥1 point) in both feet in 2 views (dorsoplantar and lateral). Although the scale description proposed in the La Trobe Foot Atlas publication describes JSN grade 3 as joint fusion, we have interpreted this more precisely as bone‐on‐bone 26.

For year 23, the presence of radiographic OA was defined as 1 radiographic feature graded as 2 or higher 24. This was limited to the dorsoplantar view to match those at year 6, when all foot radiographs had been taken of both feet together as dorsoplantar views and only available as plain film. The advice given in the La Trobe Foot Atlas indicates that use of both dorsoplantar and lateral views is the gold standard and should be applied where possible to ensure an appropriate level of sensitivity to OA 24. However, further evaluation of the La Trobe Foot Atlas has shown that good sensitivity (94.6%) can be obtained in the first MTP joint when only a dorsoplantar view is available 27. For these reasons, the first MTP joint alone was selected as the focus of investigation in this study.

All radiographs were scored by a single trained reader (PM). Reliability results for OP and JSN scoring at 5 foot joints have been detailed previously 26. Intrarater agreement was also established for the presence or absence of OA at the first MTP joint using Cohen's kappa statistic, cross‐referenced to the values criteria by Landis and Koch 28. Reliability was moderate (κ = 0.51) for the left first MTP joint and substantial (κ = 0.61) for the right first MTP joint 26.

#### Statistical analysis

Data from the Chingford 1,000 Women Study are maintained in an Access database (Microsoft). Study data for year 23 were collected and managed using research electronic data capture software 29 hosted at the University of Oxford. The data were exported, and all data evaluation and statistical analyses were conducted using Stata, version 14.1.

Complete case analysis was performed based on those participants who had radiographic foot data at both year 6 and 23 time points. Natural history was determined by observing the change in prevalence of OA in the first MTP joint of case‐matched participants from year 6 to year 23 and by defining incidence, progression, and worsening of OA in the first MTP joint during the time period of the study.

Prevalence was calculated at both the subject level (using either foot) and at the first MTP joint level (with each subject supplying 2 feet for the analysis) and was defined using the La Trobe Foot Atlas grade of ≥2 for either OPs or JSN 24. Incidence of first MTP joint OA was calculated at the subject level and was defined by having a La Trobe Foot Atlas grade of 0 or 1 of both OPs and JSN at year 6 and a grade of ≥2 for OPs or JSN at year 23. Incident unilateral and bilateral first MTP joint OA was defined as having a La Trobe Foot Atlas grade of 0 or 1 of both OPs and JSN in both first MTP joints at year 6 and having a grade of ≥2 for OPs or JSN in 1 or both first MTP joints at year 23, respectively.

Progression was calculated at the first MTP joint level and was defined as having a La Trobe Foot Atlas grade score of 2 for OPs and/or JSN in the first MTP joint at year 6, with an increase to a score of 3 for the corresponding OPs and/or JSN by year 23 in the left and right first MTP joints. At the subject level, we also determined the number of participants who had unilateral first MTP joint OA at the first period of observation (grade >2 for OPs or JSN) but bilateral disease at the second period of observation.

Worsening was calculated at the first MTP joint level and was defined as an increase of La Trobe Foot Atlas grade from any grade (including grades 0, 1, and 2). The group with worsening essentially includes incident cases, participants with disease progression, as well as participants with mild progression who moved from a La Trobe Foot Atlas grade of 0 to 1.

## RESULTS

**Figure 1:**
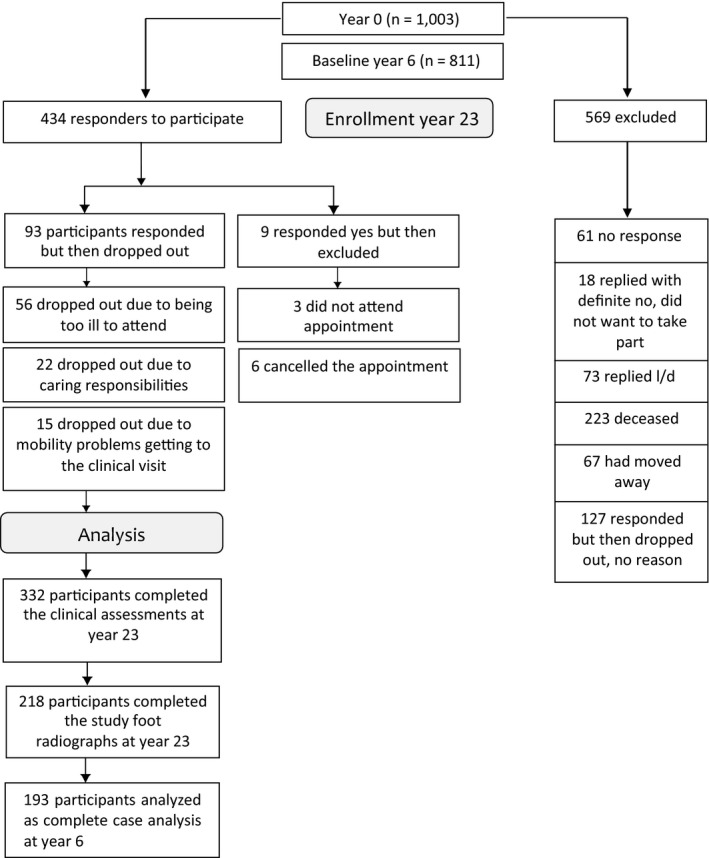
Participant recruitment flow diagram for the Chingford 1,000 Women Foot Study. l/d = long distance (the participant moved out of the area and found it too far to travel for subsequent visits); OA = osteoarthritis.

Of the original 1,003 participants at baseline (year 0), 846 (84.34%) attended the clinical appointments at year 6, and 332 (33.3%) attended clinical appointments at year 23. Reasons for loss to follow‐up from baseline included death (n = 223), withdrawal (n = 311), moved (n = 67), uncontactable (n = 61), and did not attend year 23 (n = 9) (Figure 1). There were no significant differences in terms of age, height, weight, or body mass index (BMI) between responders and nonresponders from the previous visit.

At year 23, 218 attended for foot radiographs. Of these participants, 193 had foot radiographs taken at year 6. Data from 193 participants who had complete radiographic foot data at both year 6 and year 23 were therefore included in the analyses. The mean ± SD age of included participants at year 23 was 75.7 ± 5.2 years (range 68–90 years), and the mean ± SD BMI was 27.9 ± 4.5.

#### Natural history of radiographic first MTP joint OA

In participants (n = 193) at year 6, the prevalence of first MTP joint OA in either foot was 33.2% (n = 64), being present in 21.8% (n = 42) of left feet and 24.4% (n = 47) of right feet, and being bilateral in 13.0% (n = 25). At year 23, the prevalence of first MTP joint OA in either foot had increased to 40.9% (n = 79), with 23.8% (n = 46) having involvement of the left foot, 32.6% (n = 63) the right foot, and 15.5% (n = 30) having bilateral involvement.

#### Incidence of radiographic first MTP joint OA

Of 129 participants with no OA in either first MTP joint at year 6, 7.0% of participants (n = 9) developed incident first MTP joint radiographic OA in the left foot and 17.1% (n = 22) in the right foot over a period of 19 years. At the subject level, 21.7% (n = 28) developed first MTP joint OA in either foot, and 2.3% (n = 3) developed bilateral first MTP joint OA.

#### Progression of radiographic first MTP joint OA

For participants who had first MTP joint OA defined as a score of 2 at year 6 (n = 35 left; n = 43 right), progression to a score of 3 at year 23 in the corresponding feature (OPs or JSN) was seen in 28.6% (n = 10) left first MTP joints and 34.9% (n = 15) right first MTP joints at year 23.

For participants who had bilateral first MTP joint OA, defined by a score of 2 in either JSN or OPs in both first MTP joints at year 6 (excluding those with a score of 3 in the alternate feature), progression to a score of 3 in both first MTP joints at year 23 was seen in 22.2% (n = 4) at year 23. For participants who had unilateral first MTP joint OA, defined by a score of 2 for OPs or JSN at year 6, progression to a score of 3 in both first MTP joints (bilateral OA) at year 23 was seen in 28.2% (n = 11). The data on individual OP and JSN progression can be found in Table 1.

**Table 1:** Progression of first metatarsophalangeal (MTP) joint osteoarthritis (OA) over a 19‐year period at the joint, foot, and subject level^a^

Grade 2 OA at year 6	Progression at year 23
Left JSN (n = 8)	1 (12.5)
Left OPs (n = 32)	10 (31.3)
Right JSN (n = 11)	4 (36.4)
Right OPs (n = 40)	14 (35.0)
Left first MTP joint (n = 35)	10 (28.6)
Right first MTP joint (n = 43)	15 (34.9)
Bilateral to bilateral first MTP joint progression (n = 18)	4 (22.2)
Unilateral to bilateral first MTP joint progression (n = 39)	11 (28.2)

a Values are the number (%). JSN = joint space narrowing; OPs = osteophytes.

#### Worsening of radiographic first MTP joint OA

From year 6 to year 23, for those participants who had first MTP joint JSN scores of 0, 1, or 2 (i.e., excluding any participants who had a score of 3), changes were noted toward a corresponding higher score of 1, 2, or 3 (i.e., worsening) in 28.6% (n = 54) right first MTP joints and in 33.9% (n = 63) left first MTP joints at year 23.

From year 6 to year 23, for those participants who had first MTP joint OP scores of 0, 1, or 2 (i.e., excluding any participants who had a score of 3), changes were noted toward a corresponding higher score of 1, 2, or 3 (i.e., worsening) in 30.5% (n = 58) right first MTP joints and in 23.0% (n = 43) left first MTP joints at year 23. A breakdown of worsening score changes is shown in Table 2.

**Table 2:** Worsening score changes for first metatarsophalangeal joint osteophytes (OPs) and joint space narrowing (JSN) between years 6 and 23^a^

Change in score from year 6 to 23	Right		Left	
	JSN	OPs	JSN	OPs
0–1	38 (70.4)	17 (29.3)	29 (46.0)	15 (34.9)
0–2	1 (1.9)	4 (6.9)	10 (15.9)	3 (7.0)
0–3	0 (0.0)	1 (1.7)	2 (3.2)	1 (2.3)
1–2	8 (14.8)	18 (31.0)	13 (20.6)	13 (30.2)
1–3	3 (5.6)	4 (6.9)	8 (12.7)	1 (2.3)
2–3	4 (7.4)	14 (24.1)	1 (1.6)	10 (23.3)
Total cases	54 (100.0)	58 (100.0)	63 (100.0)	43 (100.0)

a Values are the number (%).

## DISCUSSION

The results from this study provide new longitudinal evidence for the natural history of radiographic first MTP joint OA in a sample of older women from a population‐based cohort in the UK. More than one‐half of the women (59%) remained free of radiographic first MTP joint OA over the course of this study. The prevalence of first MTP joint OA increased, and 22% of those free of radiographic OA at baseline (year 6) developed it by year 23. Both progression (defined as a score of 2 at year 6 and 3 at year 23) and worsening (defined as a score of 0, 1, or 2 at year 6, increasing to higher score of 1, 2, or 3 at year 23) of OA were more evident in the right first MTP joints.

Very few studies have reported on the epidemiology of foot OA, which makes comparison of the cohort data that does exist in this field challenging 30. Figures for radiographic foot OA prevalence estimates vary considerably depending on the population, the radiographic views taken, which foot joints are examined, the grading systems applied, and whether symptomatic or asymptomatic foot OA is studied 9, 26. As such, longitudinal investigation of foot OA is rare. A recent cohort study reported little change in foot pain severity over an 18‐month period, but it used radiographic foot OA phenotypes as the baseline stratification criteria and did not perform follow‐up foot radiographs 18.

The prevalence of radiographic first MTP joint OA in our cross‐sectional analysis of data from year 6 (33.1%) and year 23 (40.9%) is higher than that for women in the Clearwater Osteoarthritis Study (17.7%), which reported a mean age of 62 years 19. This may in part be due to the differences in grading radiographic OA. The current study utilized the La Trobe Foot Atlas 24, unlike the Clearwater Osteoarthritis Study, which used Kellgren/Lawrence criteria 19, 31. Other studies using the La Trobe Foot Atlas have determined the cross‐sectional prevalence of first MTP joint OA based on the presence of corresponding symptoms and therefore report lower prevalence (7.8%) than that of radiographic change 2.

The Kellgren/Lawrence system, which is widely used to grade radiographic OA in a range of joints 31, has been criticized as having inherent difficulties at all joint sites, including the small joints of the foot, due to inconsistent interpretation and application of the grades between studies 9. To overcome these issues in inconsistent interpretation of radiographic foot OA between studies, the foot‐specific radiographic La Trobe Foot Atlas and grading system were developed 24, 27. As subsequent investigations have adopted this grading system 2, 26, 32, comparison of prevalence figures for OA with those of older investigations, such as the Clearwater study 19, has become difficult.

While just over one‐half of our participants remained free of radiographic first MTP joint OA over the course of the study, 21.7% developed first MTP joint OA in either foot, and 2.3% developed bilateral first MTP joint OA. As an indirect comparison in the same cohort, 39.5% of participants developed incident knee radiographic OA in at least 1 knee over a 14‐year period, and the annual cumulative incidence of radiographic knee OA was similar at 2.3% at the knee level and 2.8% at the subject level 17. This is supported by the findings of other investigators who reported that 2% of participants in the Framingham study had incident knee OA over a mean interval of 8.1 years 33. Incidence of hip OA is slightly higher than that reported in the knee. In the Johnston County Osteoarthritis Project, radiographic OA developed in 7.4% of the hips with no OA at baseline and 3.6% of the hips with mild or moderate OA progressed over the follow‐up period 34. Franklin et al found 2.5% to have radiographic OA of the hips with follow‐up at 11 and 28 years after the original diagnosis 35. For hand OA among women in the Framingham study, incidence is also comparable to that in the knee at 1%, which increased with age and leveled off at the age of 80 years 36, 37.

Among our participants who scored 2 on the La Trobe Foot Atlas Scale for first MTP joint OA at baseline, progression was found to be greater in the right foot, and approximately one‐third of women with unilateral first MTP joint OA at baseline developed bilateral first MTP joint OA by the end of the study. Interestingly, there was a notable difference between worsening of scores for JSN and scores for OPs in left and right feet, with right‐sided worsening being driven largely by OPs and left‐side worsening by JSN. To our knowledge, these findings have not been previously reported. Discordance in symmetry of OA has been reported for the hands, with the suggestion that biomechanical factors acting primarily through OP development may predispose patients to the asymmetric development of OA 38. In the same study, JSN was found to be more symmetrical and thus consistent with being driven more by constitutional and genetic factors. It is possible that this is also true for the foot joints. If our findings are confirmed, further work should involve exploration of risk factors such as physical and occupational activity that may contribute to increased forces and damage being transmitted through the first MTP joint. Such analysis could potentially uncover opportunities for earlier intervention and prevention of the development of first MTP joint OA.

The main limitation of our study relates to the high attrition rate of participants, which is a caveat in many longitudinal studies. There is potential for deaths and withdrawal due to disability and illness to bias the results toward a healthier cohort who attended the follow‐up visits. Also, the study population was limited to women, predominately white and older, and from a single geographic region in the UK, which limits the generalizability of the findings. Other important caveats are that only 1 radiographic view (non–weight‐bearing dorsoplantar) was examined, that our assessor was not blinded to the time point of the study at which the image was taken, and that clinical data (symptoms) and possible risk factors for progression (e.g., BMI, footwear, constitutional alignment, age at onset of OA, presence of nodal hand OA, etc.) were not examined. Such possible risk factors for progression merit investigation in future studies.

In conclusion, to our knowledge, this is the longest study to date to examine the natural history of radiographic first MTP joint OA. The findings demonstrate that the prevalence of radiographic OA within the first MTP joint in older women increases over a 19‐year period. While just over one‐half of participants remained free of radiographic first MTP joint OA during that time, incident cases, progression, and/or worsening of first MTP joint OA over time was observed in the remaining participants. The discordance in right‐left findings for OP development and JSN observed over time has not been described previously. Findings from this study implicate biomechanical factors in the development of first MTP joint OA, and investigation of potential biomechanical risk factors for the development and progression of first MTP joint OA seems warranted.

## AUTHOR CONTRIBUTIONS

All authors were involved in drafting the article or revising it critically for important intellectual content, and all authors approved the final version to be submitted for publication. Dr. Gates had full access to all of the data in the study and takes responsibility for the integrity of the data and the accuracy of the data analysis.

### Study conception and design

Bowen, Gates, McQueen, Delmestri, Drechsler, Stephensen, Doherty, Arden.

### Acquisition of data

Bowen, Gates, McQueen, Daniels, Delmestri.

### Analysis and interpretation of data

Bowen, Gates, Delmestri, Drechsler, Stephensen, Doherty, Arden.
